# Avian influenza A(H7N9) and (H5N1) infections among poultry and swine workers and the general population in Beijing, China, 2013–2015

**DOI:** 10.1038/srep33877

**Published:** 2016-09-27

**Authors:** Peng Yang, Chunna Ma, Shujuan Cui, Daitao Zhang, Weixian Shi, Yang Pan, Ying Sun, Guilan Lu, Xiaomin Peng, Jiachen Zhao, Yimeng Liu, Quanyi Wang

**Affiliations:** 1 Beijing Center for Disease Prevention and Control, Beijing, China; 2 Beijing Research Center for Preventive Medicine, Beijing, China; 3 School of Public Health, Capital Medical University, Beijing, China.

## Abstract

Although several studies have reported seroprevalences of antibody against avian influenza A(H7N9) virus among poultry workers in southern China, results have varied and data in northern China are scarce. To understand risks of H7N9 and H5N1 virus infections in northern China, a serological cohort study was conducted. Poultry workers, swine workers and the general population in Beijing, China, were evaluated through three surveys in November 2013, April 2014 and April 2015. The highest seroprevalence to H7N9 virus among poultry workers was recorded in the April 2014 and April 2015 surveys (0.4%), while that to H5N1 clade 2.3.4 or clade 2.3.2.1 virus was noted in the April 2014 survey (1.6% and 0.2%, respectively). The incidence of H7N9 virus infections among poultry workers (1.6/1000 person-months) was significantly lower than that of H5N1 clade 2.3.4 infections (3.8/1000 person-months) but higher than that of H5N1 clade 2.3.2.1 infections (0.3/1000 person-months). Compared with the general population, poultry workers were at higher risk of contracting H7N9 virus (IRR: 34.90; *p* < 0.001) or H5N1 clade 2.3.4 virus (IRR: 10.58; *p* < 0.001). Although risks of H7N9 and H5N1 virus infections remain low in Beijing, continued preventive measures are warranted for poultry workers.

In March 2013, human infections with a novel avian influenza A(H7N9) virus were discovered in Southeast China^1^,^2^. Most patients with H7N9 virus infection presented with severe lower respiratory infection and approximately one-third died^3^,^4^,^5^. Additionally, documented evidence suggested that exposure to live poultry is the most significant risk factor for infection with H7N9 virus^6^,^7^,^8^.

Along with severe and fatal cases of H7N9 virus infection reported thus far^3^,^4^,^9^, mild and asymptomatic infections in humans were also observed by surveillance^3^,^10^,^11^,^12^ and serological studies^13^,^14^,^15^,^16^. While few serological studies showed that higher seroprevalence of antibody against H7N9 virus was observed among poultry workers, compared with the general population, the results varied significantly by study^13^,^14^,^15^. Previous serological studies on H7N9 virus infection mainly utilized cross-sectional surveys, which cannot determine the incidence rate of H7N9 virus infection. Moreover, it has been difficult to compare accurately the risks of H7N9 virus infection between poultry workers and the general population because of potential recall bias and unidentified confounding factors. However, a cohort study can overcome these issues because this design can measure new infections of study participants within a defined period and related information about them is collected before the occurrence of the outcome of interest. Additionally, previous serological studies on H7N9 virus infection focused on southern China^13^,^14^,^15^, with no serological evidence reported from northern China.

In addition, given that pigs have the potential to be infected by avian influenza viruses^17^,^18^,^19^,^20^,^21^ and are considered as a “mixing vessel” of various origins of influenza viruses^22^,^23^, understanding the incidence of avian influenza virus infections in swine workers is of importance as well.

Beijing is located in northern China. Although human infections with H7N9 and H5N1 viruses have been documented in Beijing^10^,^24^, there is limited information on the prevalence of H7N9 or H5N1 viruses in poultry, pigs and humans in Beijing^25^.

To understand the risk of human infections with H7N9 and H5N1 viruses in northern China, a serological cohort study was undertaken that surveyed poultry workers, swine workers and the general population in Beijing, China, between November 2013 and April 2015.

## Results

### Participants characteristics

In November 2013, a total of 3790 participants were included in the first survey: 1258 poultry workers (33.2%), 1332 swine workers (35.1%) and 1200 general individuals (31.7%).

In April 2014, the 3790 participants in the first survey were followed up, 2563 of whom consented to participate in the second survey. Thus, the first cohort (2013–2014) included 2563 participants. To compensate for the number of participants lost to follow-up, an additional 935 participants were invited to participate in the second survey in April 2014, for a total sample size of 3498 participants.

In April 2015, the 3498 participants in the second survey were followed up, 2012 of whom consented to participate in the third survey. Thus, the second cohort (2014–2015) included 2012 participants. Similar to the second survey, an additional 1244 participants were invited to participate in the third survey to compensate for participants lost to follow-up, and 3256 participants were finally analyzed.

Statistically significant differences were observed in the participant category (*p* < 0.001) and underlying disease status (*p* < 0.001) between the three independent surveys. Additionally, a statistically significant difference was noted in the participant category (*p* < 0.001) between 2013–2014 and 2014–2015 cohorts. However, in general, similar participant characteristics were recorded among the three surveys as well as between the two cohorts (Table 1).

### Seroprevalences of antibodies to H7N9 and H5N1 viruses during the three surveys

In the first survey in November 2013, only 0.1% (1/1200) of the general population tested positive for H7N9 virus, 0.1% (1/1332) and 0.2% (2/1332) of swine workers tested positive for H5N1 clade 2.3.4 virus and H5N1 clade 2.3.2.1 virus, respectively, and no other participants tested positive for any virus. No statistically significant differences were observed in the seroprevalence of antibody to each virus between participant categories, sexes, age groups or underlying disease statuses (Table 2).

In the second survey in April 2014, 0.4% (4/1056), 1.6% (17/1056) and 0.2% (2/1056) of poultry workers tested positive for H7N9 virus, H5N1 clade 2.3.4 virus and H5N1 clade 2.3.2.1 virus, respectively. Only 0.1% (1/1254) of swine workers tested positive for H5N1 clade 2.3.4 virus. No participants from the general population tested positive for any virus. Statistically significant differences were observed in seroprevalences of antibodies to H7N9 virus (*p* = 0.008) and H5N1 clade 2.3.4 virus (*p* < 0.001) between participant categories. In addition, a statistically significant difference in H5N1 clade 2.3.4 antibody seroprevalence existed between males and females (*p* = 0.007) (Table 2).

In the third survey in April 2015, 0.4% (4/1123), 0.2% (2/1123) and 0.1% (1/1123) of poultry workers tested positive for H7N9 virus, H5N1 clade 2.3.4 virus and H5N1 clade 2.3.2.1 virus, respectively. Among swine workers, 0.1% (1/998) and 0.2% (2/998) tested positive for H5N1 clade 2.3.4 virus and H5N1 clade 2.3.2.1 virus, respectively. No participants from the general population tested positive for any virus. A statistically significant difference was observed in the seroprevalence of antibody to H7N9 virus (*p* = 0.023) between participant categories. However, no statistically significant differences in the seroprevalence of antibody to each virus existed between sexes, age groups or underlying disease statuses (Table 2).

For poultry workers, there was a statistically significant difference in the seroprevalence of antibody to H5N1 clade 2.3.4 virus among the three surveys (*p* < 0.001), but not in that to H7N9 or H5N1 clade 2.3.2.1 virus. For either swine workers or the general population, no statistically significant difference in the seroprevalence of antibody to each virus among the three surveys was observed.

### Incidence of H7N9 and H5N1 virus infections in the 2013–2014 and 2014–2015 cohorts

In the 2013**–**2014 and 2014**–**2015 cohorts, the overall incidence density rate of H7N9 virus infections (0.4/1000 person-months) was significantly lower than that of H5N1 clade 2.3.4 virus infections (1.3/1000 person-months, *p* < 0.001), and similar to that of H5N1 clade 2.3.2.1 virus infections (0.2/1000 person-months, *p* = 0.068). Furthermore, among poultry workers, the incidence density rate of H7N9 virus infections (1.6/1000 person-months) was significantly lower than that of H5N1 clade 2.3.4 virus infections (3.8/1000 person-months, *p* = 0.004), but significantly higher than that of H5N1 clade 2.3.2.1 virus infections (0.3/1000 person-months, *p* = 0.008) (Table 3).

After adjusting for sex, age group and underlying disease status, multivariate Poisson regression analysis showed that compared with the general population, poultry workers were more likely to have infection with H7N9 virus (incidence rate ratio [IRR]: 34.90, 95% CI: 7.47–∞; *p* < 0.001) or H5N1 clade 2.3.4 virus (IRR: 10.58, 95% CI: 4.43–25.30; *p* < 0.001). However, neither poultry workers nor swine workers were at a higher risk of H5N1 clade 2.3.2.1 virus infection compared with the general population (Table 3).

## Discussion

This study found that the risk of H7N9 and H5N1 virus infections among poultry and swine workers and the general population in Beijing, China remained low since 2013, as indicated by the seroprevalences of antibodies in three cross-sectional surveys and the incidence rates of infections in the cohorts. Interestingly, this study revealed that poultry workers, rather than swine workers, were at a higher risk of contracting H7N9 and H5N1 clade 2.3.4 viruses compared with the general population.

Previous serological studies regarding human H7N9 virus infection have mainly utilized cross-sectional surveys; however, a cohort design, which has advantages for examining avian influenza virus infection, was used in this study. A cross-sectional survey can only determine whether participants have prior viral infections; it fails to detect the exact time of infection. However, a cohort study can calculate the incidence rate of infection, which reflects the number of infections in a given period of time. In addition, the accuracy of comparing the risk of virus infections between various populations may be influenced by recall bias and unidentified confounding factors in a cross-sectional survey, but a cohort study can overcome this issue because the related information about study participants is collected before the occurrence of the outcome of interest.

The incidence rate of H7N9 virus infections among poultry workers in Beijing during the study period (November 2013 to April 2015; 17 months) was much lower than that in Shenzhen from May to December, 2013 (7 months)^14^. In addition, none of poultry workers were seropositive for H7N9 virus in Beijing in November 2013, while a slight increase in the seroprevalence of antibodies to H7N9 virus was observed in the subsequent surveys in April 2014 (0.4%) and April 2015 (0.4%). However, much higher seroprevalences of antibody against H7N9 virus among poultry workers were reported in other studies conducted in southern China^13^,^14^,^15^. The lower seroprevalence of antibody against H7N9 virus and incidence rate of infections among poultry workers in Beijing compared with those in southern China might be a result of increased circulation of H7N9 virus in southern provinces, as demonstrated by a greater number of reported H7N9 cases and potential H7N9-positive markets in southern provinces^26^,^27^. Variation in the serological results might also result from participant differences. Our study did not include live poultry market workers because live poultry markets in Beijing have been closed since 2005^24^; however, participants enrolled in previous serological studies on H7N9 avian influenza included live poultry market workers. The study conducted in Guangdong Province showed that the seroprevalence of antibody to H7N9 virus in poultry market workers was much higher than that of poultry farm workers^15^. In addition, extremely low seroprevalences of antibody to H7N9 virus in the general population and swine workers were found in this study, similar to the results of serological studies in southern China^13^,^14^,^15^.

In this study, the seroprevalences of antibodies to H5N1 clade 2.3.4 or clade 2.3.2.1 virus in poultry workers were very low in the three cross-sectional surveys, and similar findings were also reported in studies conducted in southern China during the similar period^14^,^15^ as well as a study conducted in Beijing in 2011^25^. Additionally, none of the general population participants had antibodies to H5N1 clade 2.3.4 or clade 2.3.2.1 virus in this study, similar to the findings of other reports^14^,^15^.

In Beijing, an increased number of laboratory-confirmed patients of H7N9 virus infection has been reported in recent years^10^,^24^,^26^; however, this serological study showed that the incidence rate of H5N1 clade 2.3.4 virus infection was significantly higher than that of H7N9 virus infection in Beijing between November 2013 and April 2015. This finding indicated that although H7N9 virus was considered to pose a greater pandemic threat than H5N1 virus^26^, close attention to the risk of H5 virus infection is necessary, particularly in the context of the recent emergence and epidemic of H5 clade 2.3.4.4 viruses^28^.

In this study, we used a prospective cohort study design to establish that poultry workers were at a higher risk of H7N9 or H5N1 clade 2.3.4 virus infection compared with the general population, as described by previous cross-sectional studies^15^. A similar trend was not observed in swine workers. Hence, we recommend that poultry workers use personal protective equipment and receive seasonal influenza vaccination to reduce the risk of avian influenza virus infection, as well as potential reassortment of avian influenza and seasonal influenza viruses.

Our study has several limitations. First, the incidence of H7N9 and H5N1 virus infections relied on seroconversion of antibodies against respective viruses; however, we did not determine the incidence of symptomatic infections confirmed by virological assays. Second, although post-infection ferret antiserum against A/Anhui/1/2005 (H5N1) (clade 2.3.4) has very low cross-reactivity with H5N6 or H5N8 virus^29^, suggesting that antibodies against H5N1 viruses detected by the HI assay in this study are specific to H5N1 viruses, we cannot exclude the possibility that these antibodies might be elicited by other H5 viruses. Moreover, antibodies against H7N9 virus detected in this study might be elicited by infections with other H7 viruses. Third, more poultry and swine workers were lost to follow-up than general population participants, which might be because of the high turnover rate among occupational populations in Beijing, as most are migrant workers from other provinces.

The risks of H7N9 and H5N1 virus infection remain low in poultry workers, swine workers and the general populations in Beijing, China. Importantly, the risk posed by H5 viruses appears not lower than that of H7N9 viruses. Continued surveillance, using personal protective equipment and seasonal influenza vaccination are warranted for poultry workers, as they demonstrated a higher risk of H7N9 and H5N1 virus infection.

## Methods

### Ethics statement

This study was approved by the institutional review board and human research ethics committee of Beijing Center for Disease Prevention and Control, and all methods were carried out in accordance with the principles of the Declaration of Helsinki. Informed consent was obtained from all participants.

### Participants and study design

This serological cohort study enrolled poultry workers, swine workers and individuals in the general population in Beijing, China and comprised three serological surveys implemented in November 2013, April 2014 and April 2015. For the first survey conducted in November 2013, multi-stage cluster sampling was used to enroll poultry and swine workers, and stratified multi-stage random sampling was used to recruit general population participants.

Poultry and swine workers included workers from commercial breeding farms, abattoirs and private breeding sites. First, five districts that contain poultry and swine-related industries were selected. Second, for poultry and swine workers from commercial breeding farms and abattoirs, two poultry and two swine commercial breeding farms as well as the same number of poultry and swine abattoirs were selected in each of the five districts, and all poultry and swine workers from the selected commercial breeding farms and abattoirs were invited to participate in this study. For workers from private breeding sites, two towns with poultry industries and two towns with swine industries were selected in each of the five districts, and all workers from private poultry/swine-breeding sites in the selected towns were invited to participate in this study.

The general population was classified as participants not involved in poultry and swine-related careers, and recruited from the same districts as poultry and swine workers. The sampling frame is briefly described as follows. First, one town and one street were randomly selected in each of the five districts. Second, two villages were randomly selected from the town chosen in the first stage, and two communities were selected from the street. Third, 60 people aged ≥18 years were randomly selected from each village or street.

The participants enrolled in the first survey in November 2013 were invited to complete a questionnaire and provide serum samples for antibody detection. For the second survey conducted in April 2014, individuals who participated in the first survey were followed up to provide the second serum samples for antibody detection. However, as some participants were lost to follow-up, other individuals with similar occupations and residences as those lost to follow-up were invited to participate in the second survey in April 2014 to compensate for the lost sample size as much as possible,. For the third survey conducted in April 2015, individuals who participated in the second survey were followed up to provide serum samples for antibody detection. As in the second survey, individuals with similar characteristics as those lost to follow-up were invited to participate in the third survey.

Individuals who participated in the first and second surveys were referred to as the 2013–2014 cohort, and those who participated in the second and third surveys were referred to as the 2014–2015 cohort.

After informed consent was obtained from the participants, a questionnaire was administered via a face-to-face interview by trained staff. The questionnaire collected information on occupation, sex, age, underlying conditions and exposure to birds or pigs. Blood samples were collected from the participants for antibody testing against H7N9 and H5N1 viruses.

### Laboratory Testing

Serum samples were pretreated and assayed by the hemagglutination-inhibition (HI) assay, as previously described^30^. Serum samples were treated with receptor destroying enzyme (RDE) and absorbed with horse erythrocytes. A 1:10 dilution was prepared for each pre-treated serum sample to test for specific antibodies against H7N9 and H5N1 virus antigens using 1% horse erythrocytes. H7N9 and H5N1 virus antigens used for HI assay were the vaccine strains of A/Anhui/1/2013 (H7N9) (NIBRG-268), A/Anhui/01/2005 (H5N1)-PR8-IBCDC-RG5 (clade 2.3.4) and A/Hubei/1/2010 (H5N1) (clade 2.3.2.1). Serum samples with a 1:10 dilution that were able to inhibit virus-induced hemagglutination were then serially diluted (1:10, 1:20, 1:40, 1:80, 1:160, 1:320, 1:640 and 1:1280) for the HI assay. The HI titer was calculated as the reciprocal of the highest dilution of serum that inhibited virus-induced hemagglutination of the horse erythrocytes. A titer of 1:80 was considered as positive^13^. In addition, seroconversion of antibody against H7N9 or H5N1 virus was considered as infection, and defined as a 4-fold or greater increase in antibody HI titer between paired serum specimens with a titer ≥1:40 for the second specimen^14^.

### Statistical analysis

Data were entered in duplicate using EpiData Software and analyzed using SAS^®^ University Edition (SAS Institute, Cary, NC, USA). We estimated the seroprevalence rates in three independent surveys and calculated the person-time incidence rates indicated by serologically confirmed infections (seroconversion) per 1000 person-months as follows: total number of infections in both cohorts divided by the number of person-months of follow-up. Percentages were calculated for categorical variables. Subgroup comparisons of participant characteristics were compared using Pearson’s χ^2^ test. Seroprevalence rates of H7N9 or H5N1 virus infection (HI titer ≥1:80) were compared between subgroups using Pearson’s χ^2^ test or Fisher’s exact test. Multivariate Poisson regression models were used to compare the person-month incidence rates of H7N9 and H5N1 virus infections between participant categories in the cohorts after adjusting for sex, age group and underlying disease status, assessed by the incidence rate ratio (IRR)^31^. Concerning zero infection in certain groups, exact conditional analysis was used to estimate the IRR and 95% CI. All tests were two-sided, and statistical significance was defined as *p* < 0.05.

## Additional Information

**How to cite this article**: Yang, P. *et al*. Avian influenza A(H7N9) and (H5N1) infections among poultry and swine workers and the general population in Beijing, China, 2013–2015. *Sci. Rep*. **6**, 33877; doi: 10.1038/srep33877 (2016).

## Figures and Tables

**Table 1:** Characteristics of the participants in three cross-sectional surveys in 2013–2015 and the respective cohorts in 2013–2014 and 2014–2015.

Characteristic	Cross-sectional surveys			*P* value*	Cohorts (Year)		*P* value*
	November 2013	April 2014	April 2015		2013–2014 (Nov 2013–Apr 2014)	2014–2015 (Apr 2014–Apr 2015)	
Participant category				<0.001			<0.001
Poultry workers	1258 (33.2)	1056 (30.2)	1123 (34.5)		791 (30.9)	512 (25.4)	
Swine workers	1332 (35.1)	1254 (35.8)	998 (30.7)		862 (33.6)	568 (28.2)	
General population	1200 (31.7)	1188(34.0)	1135 (34.9)		910 (35.5)	932 (46.3)	
Sex				0.051			0.529
Male	1933 (51.0)	1686 (48.3)	1595 (49.0)		1224 (47.8)	941 (46.8)	
Female	1855 (49.0)	1807 (51.7)	1658 (51.0)		1338 (52.2)	1068 (53.2)	
Age group				0.907			0.152
<50 years	2200 (58.1)	2018 (57.8)	1871 (57.6)		1435 (56.1)	1085 (54.0)	
≥50 years	1585 (41.9)	1471 (42. 2)	1377 (42.4)		1124 (43.9)	926 (46.0)	
Having underlying disease				<0.001			0.121
No	3489 (92.1)	3278 (93.7)	2950 (90.6)		2349 (91.7)	1868 (92.9)	
Yes	301 (7.9)	219 (6.3)	305 (9.4)		214 (8.3)	143 (7.1)	
Total	3790 (100)	3498 (100)	3256 (100)	—	2563 (100)	2012 (100)	—

Note. Data are expressed as proportion (%) of participants, unless otherwise indicated.

*Compared using Pearson’s χ^2^ test.

**Table 2:** Seroprevalences of antibodies (HI titer ≥1: 80) to H7N9 and H5N1 viruses in poultry workers, swine workers and the general population in three cross-sectional surveys (2013–2015).

Period	Characteristics	H7N9	*p* value	H5N1 clade 2.3.4	*P* value	H5N1 clade 2.3.2.1	*p* value
November 2013	Participant category						
	Poultry workers	0/1258 (0)	0.317^‡^	0/1258 (0)	1.000^‡^	0/1258 (0)	0.334^‡^
	Swine workers	0/1332 (0)		1/1332 (0.1)		2/1332 (0.2)	
	General population	1/1200 (0.1)		0/1200 (0)		0/1200 (0)	
	Sex						
	Male	0/1933 (0)	0.490^‡^	1/1933 (0.1)	1.000^‡^	2/1933 (0.1)	0.166^‡^
	Female	1/1855 (0.1)		0/1855 (0)		0/1855 (0)	
	Age group						
	<50 years	1/2200 (0.0)	1.000^‡^	1/2200 (0.0)	1.000^‡^	2/2200 (0.1)	0.513^‡^
	≥50 years	0/1585 (0)		0/1585 (0)		0/1585 (0)	
	Having underlying disease						
	No	1/3489 (0.0)	1.000^‡^	1/3489 (0.0)	1.000^‡^	2/3489 (0.1)	1.000^‡^
	Yes	0/301 (0)		0/301 (0)		0/301 (0)	
April 2014	Participant category						
	Poultry workers	4/1056 (0.4)	0.008^‡^	17/1056 (1.6)	<0.001*	2/1056 (0.2)	0.091^‡^
	Swine workers	0/1254 (0)		1/1254 (0.1)		0/1254 (0)	
	General population	0/1188 (0)		0/1188 (0)		0/1188 (0)	
	Sex						
	Male	2/1686 (0.1)	1.000^‡^	3/1686 (0.2)	0.007*	1/1686 (0.1)	1.000^‡^
	Female	2/1807 (0.1)		15/1807 (0.8)		1/1807 (0.1)	
	Age group						
	<50 years	3/2018 (0.1)	0.643^‡^	12/2018 (0.6)	0.447*	0/2018 (0)	0.178^‡^
	≥50 years	1/1471 (0.1)		6/1471 (0.4)		2/1471 (0.1)	
	Having underlying disease						
	No	4/3278 (0.1)	1.000^‡^	17/3278 (0.5)	1.000^‡^	2/3278 (0.1)	1.000^‡^
	Yes	0/219 (0)		1/219 (0.5)		0/219 (0)	
April 2015	Participant category						
	Poultry workers	4/1123 (0.4)	0.023^‡^	2/1123 (0.2)	0.417^‡^	1/1123 (0.1)	0.209^‡^
	Swine workers	0/998 (0)		1/998 (0.1)		2/998 (0.2)	
	General population	0/1135 (0)		0/1135 (0)		0/1135 (0)	
	Sex						
	Male	1/1595 (0.1)	0.625^‡^	0/1595 (0)	0.250^‡^	1/1595 (0.1)	1.000^‡^
	Female	3/1658 (0.2)		3/1658 (0.2)		2/1658 (0.1)	
	Age group						
	<50 years	2/1871 (0.1)	1.000^‡^	2/1871 (0.1)	1.000^‡^	3/1871 (0.2)	0.267^‡^
	≥50 years	2/1377 (0.1)		1/1377 (0.1)		0/1377 (0)	
	Having underlying disease						
	No	4/2950 (0.1)	1.000^‡^	3/2950 (0.1)	1.000^‡^	3/2950 (0.1)	1.000^‡^
	Yes	0/305 (0)		0/305 (0)		0/305 (0)	

Note. Data are expressed as proportion (%) of participants, unless otherwise indicated.

*Compared using Pearson’s χ^2^ test.

^‡^Compared using Fisher’s exact test.

**Table 3:** Risk of H7N9 and H5N1 virus infections in the 2013–2014 and 2014–2015 cohorts.

Virus type	Participant category	Number of infections^†^/person-months (‰)	Unadjusted IRR (95% CI), p value^*^	Adjusted IRR (95% CI), *p value*^#^
H7N9	Poultry workers	16/10099 (1.6)	35.13 (7.55–∞)^‡^ <*0*.*001*	34.90 (7.47–∞)^‡^ <*0*.*001*
	Swine workers	0/11126 (0)	NA	NA
	General population	0/15734 (0)	Reference	Reference
	Overall	16/36959 (0.4)		
H5N1 clade 2.3.4	Poultry workers	38/10099 (3.8)	9.85 (4.17–23.31) <*0*.*001*	10.58 (4.43–25.30) <*0*.*001*
	Swine workers	4/11126 (0.4)	0.94 (0.27–3.34) *0*.*926*	1.15 (0.32–4.11) *0*.*833*
	General population	6/15734 (0.4)	Reference	Reference
	Overall	48/36959 (1.3)		
H5N1 clade 2.3.2.1	Poultry workers	3/10099 (0.3)	2.33 (0.39–13.96) *0*.*353*	2.06 (0.34–12.43) 0.432
	Swine workers	2/11126 (0.2)	1.41 (0.20–10.03) *0*.*730*	1.34 (0.19–9.55) 0.769
	General population	2/15734 (0.1)	Reference	Reference
	Overall	7/36959 (0.2)		

Note. IRR: incidence rate ratio; CI: confidence interval; NA: not available.

^†^Seroconversion of antibody against H7N9 or H5N1 virus was considered as infection, and defined as a 4-fold or greater increase in antibody titer by hemagglutination-inhibition assay between paired serum specimens with a titer ≥1:40 for the second specimen.

*Univariate Poisson regression model was used to compare person-month incidence rates of H7N9 or H5N1 virus infections between participant categories.

^#^Multivariate Poisson regression model was used to compare person-month incidence rates of H7N9 or H5N1 virus infections between participant categories after adjusting for sex, age group and underlying disease status.

^‡^Exact conditional analysis was used to estimate the IRRs and 95% CIs.
